# Pan-Cancer Classification Based on Self-Normalizing Neural Networks and Feature Selection

**DOI:** 10.3389/fbioe.2020.00766

**Published:** 2020-08-04

**Authors:** Junyi Li, Qingzhe Xu, Mingxiao Wu, Tao Huang, Yadong Wang

**Affiliations:** ^1^Department of Computer Science and Technology, Harbin Institute of Technology, Shenzhen, China; ^2^Shanghai Institute of Nutrition and Health, Shanghai Institutes for Biological Sciences, Chinese Academy of Sciences, Shanghai, China

**Keywords:** cancer classification, pan-cancer, self-normalizing neural network, copy number variation, feature selection

## Abstract

Cancer is a one of the severest diseases and cancer classification plays an important role in cancer diagnosis and treatment. Some different cancers even have similar molecular features such as DNA copy number variant. Pan-cancer classification is still non-trivial at molecular level. Herein, we propose a computational method to classify cancer types by using the self-normalizing neural network (SNN) for analyzing pan-cancer copy number variation data. Since the dimension of the copy number variation features is high, the Monte Carlo feature selection method was used to rank these features. Then a classifier was built by SNN and feature selection method to select features. Three thousand six hundred ninety-four features were chosen for the prediction model, which yields the accuracy value is 0.798 and macro F1 is 0.789. We compared our model to random forest method. Results show the accuracy and macro F1 obtained by our classifier are higher than those obtained by random forest classifier, indicating the good predictive power of our method in distinguishing four different cancer types. This method is also extendable to pan-cancer classification for other molecular features.

## Background

Cancer is a one of the severest diseases which cause abnormal cell growths or tumors that metastasize to other parts of human body (Mayer et al., 2017). There are around 8 million human deaths related to cancer each year (Wild et al., 2014). Cancer classification is important for cancer diagnosis and drug discovery and can help improving treatment of patients and their life quality (Lu and Han, 2003). To decrease the effect of cancer to human health, tremendous research has been done to the cancer diagnosis and treatment, among which molecular-feature-based cancer classification is an important perspective. Due to the drop in the cost of sequencing technology in recent years, the output of sequencing data has increased dramatically. This provides adequate data for cancer analysis. Copy number variance (CNV) has also been shown to be associated with different cancers (Greenman et al., 2007; Wang et al., 2013). Some different cancers even have similar CNV patterns and mechanisms (Hoadley et al., 2018). We focus on CNV data analysis in this study. We aim to find out an applicable computational method to classify different cancer types. At present, some machine learning models are widely used in data analysis. Some models have been used to analyze the CNV data for cancer analysis (Ostrovnaya et al., 2010; Ding et al., 2014). The utility of machine learning in revealing relationships between recurrent constitutional CNVs and cancers shows CNV data analysis is applicable to multi-type of cancers with a significant molecular component.

Deep learning has recently been widely used in computational scientific areas such as computer vision, natural language processing, computational biology (LeCun et al., 2015; Najafabadi et al., 2015; Angermueller et al., 2016; Sultana et al., 2020). The essence of deep learning algorithms is the domain independent idea of using hierarchical layers of learned abstraction to efficiently accomplish a complicated task. It uses many layers of convolutional or recurrent neural networks. The feed-forward neural network (FNN) is suitable for data without sequential features. However, there are some drawbacks of the FNNs. For instance, internal covariate shift (Ioffe and Szegedy, 2015) might causes the low training speed and poor generalization (Bengio et al., 1994; Pascanu et al., 2012, 2013). FNN might leads to invalid gradient too (Klambauer et al., 2017). Therefore, normalization is used and the self-normalizing neural network (SNN) (Klambauer et al., 2017) is proposed to overcome these short backs. SNNs make it possible for deep network applications on general data such as sequencing CNV data and SNNs have yielded the best results on some drug discovery and astronomy tasks.

In this study, we use a SNN-based prediction model to classify and analyze cancer patients with four cancers (LUAD, OV, LIHC, and BRCA). The data we used come from CNV data of The Cancer Genome Atlas (TCGA) (Grossman et al., 2016). We integrate a method which was used by Pan et al. (2018) to identify atrioventricular septal defect in Down syndrome patients to build our prediction model. Since the CNV data has a very high dimension, feature selection method is applied to identify important CNV features. Then a deep SNN model is trained based on these CNV features to perform pan-cancer classification. The normally used classification algorithm random forest (Cutler et al., 2012) is also used to compare with our model for its predictive ability in four different types of patient samples.

## Methods

### Data Retrieval and Preprocessing

We download and collate the copy number variation data of 518 Lung adenocarcinoma (LUAD) patients, 597 Ovarian serous cystadenocarcinoma (OV) patients, 372 Hepatocellular carcinoma (LIHC) patients and 597 Breast cancer (BRCA) patients from TCGA database (Grossman et al., 2016), including the information of the copy number variation of probes. We use GISTIC2.0 (Mermel et al., 2011) to analyze the data. GISTIC2.0 can identify the key drivers of somatic copy number alterations (SCNAs) by the frequency and magnitude of mutation events. By using GISTIC2.0, we can select more important copy number variant genes, and then model the molecular information data of cancer patients more precisely. From the result generated form GISTIC2.0, we get a table which has 23,109 features. A series of discrete values is used to represent the specific type of copy number variation.

### Approach for Cancer Classification

#### Feature Analysis

Since the dimensions of the CNV features are high, in order to avoid over-fitting, we need to select some features that can effectively classify patients. Therefore, we employed Monte Carlo Feature Selection (MCFS) (Draminski et al., 2008) and Incremental Feature Selection (IFS) methods as we used these two method before (Pan et al., 2018).

Monte Carlo Feature Selection method is proposed to improve a feature ranking obtained from an ensemble of decision trees. The general idea is to select *s* subset of the original *d* features, each with *m* features randomly selected. We repeat the selection process for s times, so that *s* feature subsets and a total of *t* × *s* tree classifier was obtained. Each feature *f* is assigned a score called relative importance (*RI*_*f*_) which is assigned greater to feature *f* if it contributes more in the classification using the tree classifiers. *RI* of *f* is estimated by the Equation (1):

(1) $$\begin{array}{l} {RI}_{f}\;=\;\sum_{\tau =1}^{s^{*}t}{\left(wAcc\right)}^{u}\;\sum_{n_{f}\left(\tau \right)}IG\left(n_{f}\left(\tau \right)\right)\;{\left(\frac{no.\;in\;n_{f}\left(\tau \right)}{no.\;in\;\tau }\right)}^{v} \end{array}$$

***wAcc*** is the weighted accuracy and ***IG***(***n***_***f***_(***τ***)) is the information gain of node ***n***_***f***_(***τ***). no.in ***n***_***f***_(***τ***) is the number of patients in ***n***_***f***_(***τ***) and no.in ***τ*** is the number of patients in tree ***τ***. *u* and *v* are a fixed real number.

The ***wAcc*** is defined by Draminski as Equation (2):

(2) $$\begin{array}{l} wAcc=\;\frac{1}{c}\;\sum_{i\;=\;1}^{c}\frac{n_{ii}}{n_{i1}+n_{i2}+\ldots +n_{ic}} \end{array}$$

In Equation (2), c is the number of classes and ***n***_***ij***_ is the number of patients from class i that are classified as class j. The ***IG***(***n***_***f***_(***τ***)) is defined by Equation (3):

(3) $$\begin{array}{l} IG\left(n_{f}\left(\tau \right)\right)=Entropy\left(T\right)-Entropy\left(T,\;f\right) \end{array}$$

In Equation (3), T is the class label of node ***n***_***f***_(***τ***), Entropy(T) is the entropy of the frequency table of T and Entropy (*T, f*) is the entropy of the frequency table of the two variables T and f.

We used the MCFS method of Draminski and obtained a ranked feature list according to their RI values evaluate by the algorithm, which can be defined as Equation (4).

(4) $$\begin{array}{l} F=\left[f_{1},\;f_{2},\;\ldots \;,\;f_{M}\right] \end{array}$$

And in Equation (4) M means the 23,109 CNV features.

Then we aimed to select a subgroup of CNV features to build a classification model. Therefore, in order to avoid training all CNV feature sets, we used Incremental Feature Selection method on previous obtained feature list. We first determine the approximate feature interval from which we can find optimal features. We defined CNV feature subsets as $S_{1}^{1},S_{2}^{1},\ldots ,S_{l}^{1}$, where $S_{i}^{1}=f_{1},f_{2},\ldots ,f_{i^{*}k}$, i.e., and the *i*th feature subset had the first i times k features in the original M CNV feature list. Classification model was built by using features in each feature subset of corresponding patient samples in dataset. To estimate the CNV feature interval, we tested performances of different classification model based on different subsets. The feature subset was selected when it had the best performance.

#### Classification Methods

We need an algorithm to classify pan-cancer patients based on the selected subset of CNV features. Here, neural network SNN was used and RF method was applied for comparison.

(a) Self-Normalizing Neural Network Algorithm

SNN is proposed to enable high-level abstract representations through keeping neuron activations converge toward zero mean and unit variance (Klambauer et al., 2019). Klambauer et al. proposed a Scale ELU (SELU) function as activation function.

(5) $$\begin{array}{l} selu\left(x\right)=\;\lambda \;\{\begin{array}{c} x,\;x>0\\ {\alpha e}^{x}-\;\alpha ,\;x\leq 0 \end{array} \end{array}$$

where scale λ= 1.0507 and α = 1.6733 (see Klambauer et al., 2017 for details on the derivation of these two parameters).

By using the Banach fixed-point theorem, Klambauer et al. prove that activations close to zero mean and unit variance that are propagated through many network layers will converge toward zero mean and unit variance. A specific method to initialize SNNs and alpha dropout (Klambauer et al., 2017) are also proposed to make SNNs have a fixed point at zero mean and unit variance. In this study, the SNN classifiers those we constructed have three hidden layers with 200 hidden nodes of each layer.

(b) Random Forest Algorithm

The random forest (RF) method is a supervised classification and regression algorithm (Cutler et al., 2012). The RF method builds multiple decision trees and merges them together to get a more accurate prediction. It adds additional randomness to the model when it growing the trees. Instead of searching for the most important feature when splitting a node, it searches for the best feature among a random subset of features. This generally results in a better model. The RF method has been widely used in machine learning area and is applied here to compare our model.

#### Performance Evaluation

Since pan-cancer classification is a multi-classification problem, we use accuracy (ACC) to measure the performance. There are also precision and recall to measure performance in a binary classification problem. One measurement closely related to these two values is F-score, which is a comprehensive indicator of precision and recall. That means, F-score is a parameter used to adjust the ratio of these two parts. When this parameter is 1, it degenerates into a harmonic average called F1-score. The multi-classification evaluation was split into multiple binary classification problems, and each F1-score was calculated. The average of the F1 scores was defined as Macro F1. To evaluate prediction of SNN classifier, we performed a 10-fold cross-validation (Kohavi, 1995; Chen et al., 2017, 2018).

## Results

To evaluate the best features for discriminating four types of cancer samples, a MCFS method was used to rank all features according to their RI values by using Monte Carlo method and decision trees. We selected the top 5,000 CNV features and applied IFS method.

**Figure 1:**
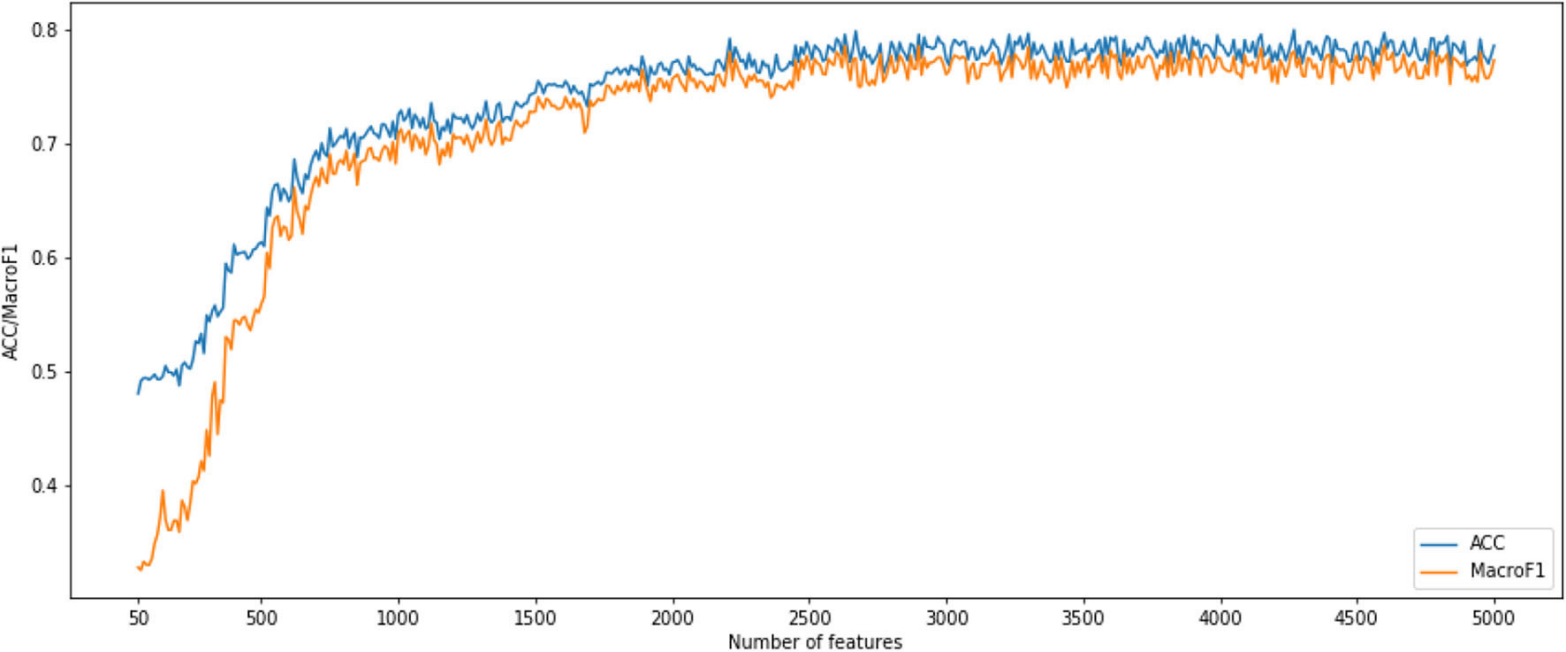
Incremental feature selection (IFS) curves derived from the IFS method and SNN algorithm. IFS curve with *X*-values from 50 to 5,000.

After using MCFS for CNV feature sorting, we obtained two feature subset series. For the first CNV feature subsets, the parameter *k* is set to 10. That means, the *i*-th feature subset contains the first 10 times *i* features in the original CNV feature list. We constructed an SNN-based classification model on each feature subset, performed a 10-fold cross-validation and calculated its accuracy and macro F1 values. To show the changes of accuracy and macro F1 values, an IFS curve was generated as Figure 1. In Figure 1, the accuracy and macro f1 values are the Y axis and the number of features is the X axis. Both curves become stable after number of features >2,500 and them reached acceptable values. Therefore, we selected the number interval as [2,500, 4,999] for classifier to select the best number of features.

**Figure 2:**
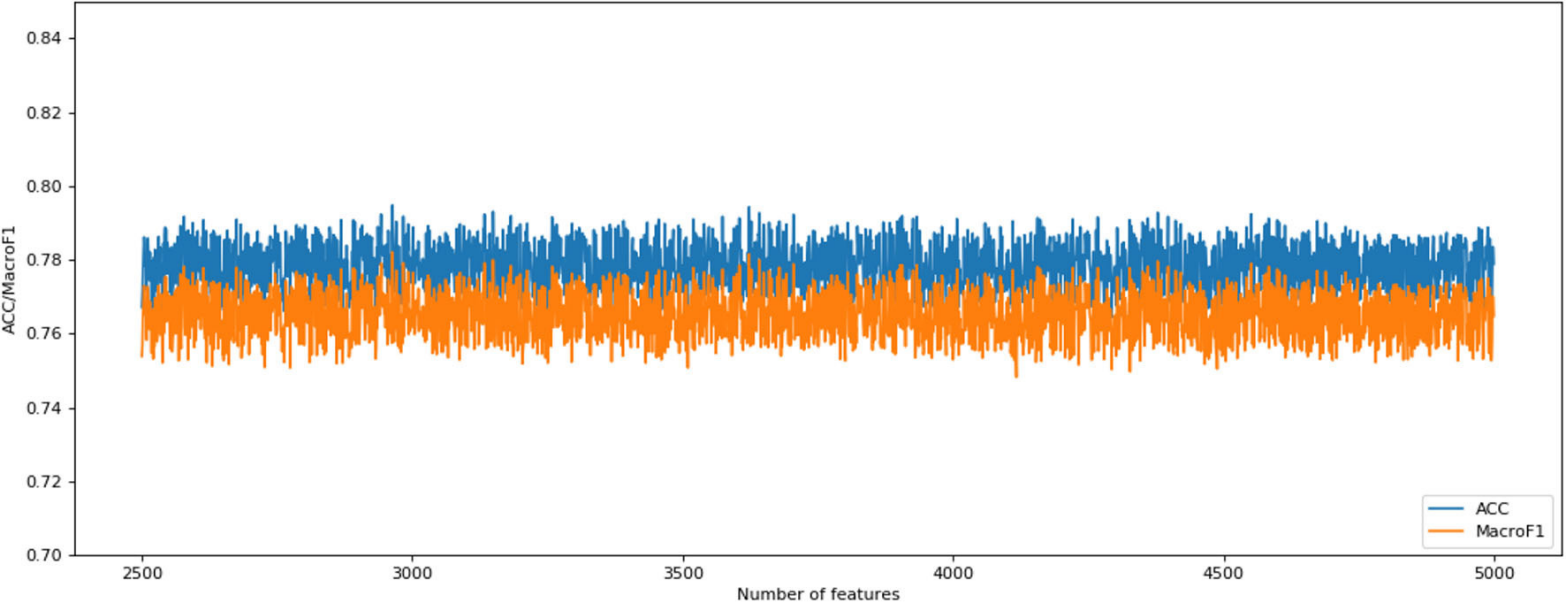
Incremental feature selection (IFS) curves derived from the IFS method and SNN algorithm. IFS curve with X-values of 2501–4,999 for SNN algorithm.

**Figure 3:**
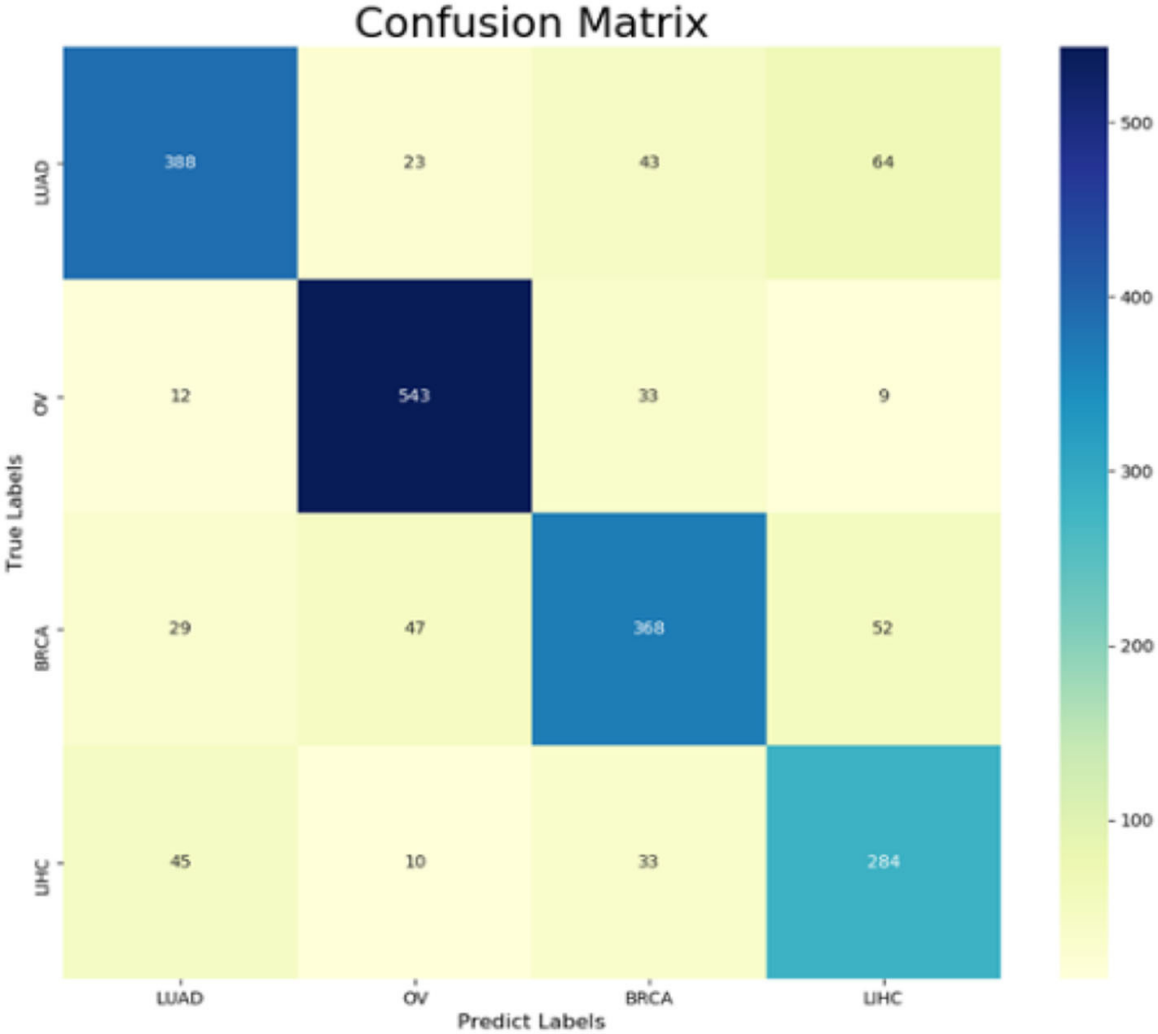
Confusion matrix from pan-cancer classification by using SNN and features selection.

The following CNV feature subset is constructed by using the number of features in the number interval [2,500, 4,999]. By testing all of these subsets, we obtained the corresponding accuracy and macro F1 values. We also plotted the IFS curves to show these values in Figure 2. The best accuracy and macro F1 values were generated when using the first 3,694 features to construct the SNN-based classification model. Thus, these first 3,694 genes were select for the final model. In the meantime, we used RF method as a comparison. The RF generated accuracy and macro F1 are much lower than the SNN one, which proves the efficiency of the deep SNN classifier. Therefore, we obtained the best feature subset and the optimal SNN-based model. Its ACC is 0.798 and the corresponding macro F1 is 0.789. Figure 3 is confusion matrix and shows the good classification result from our model.

**Figure 4:**
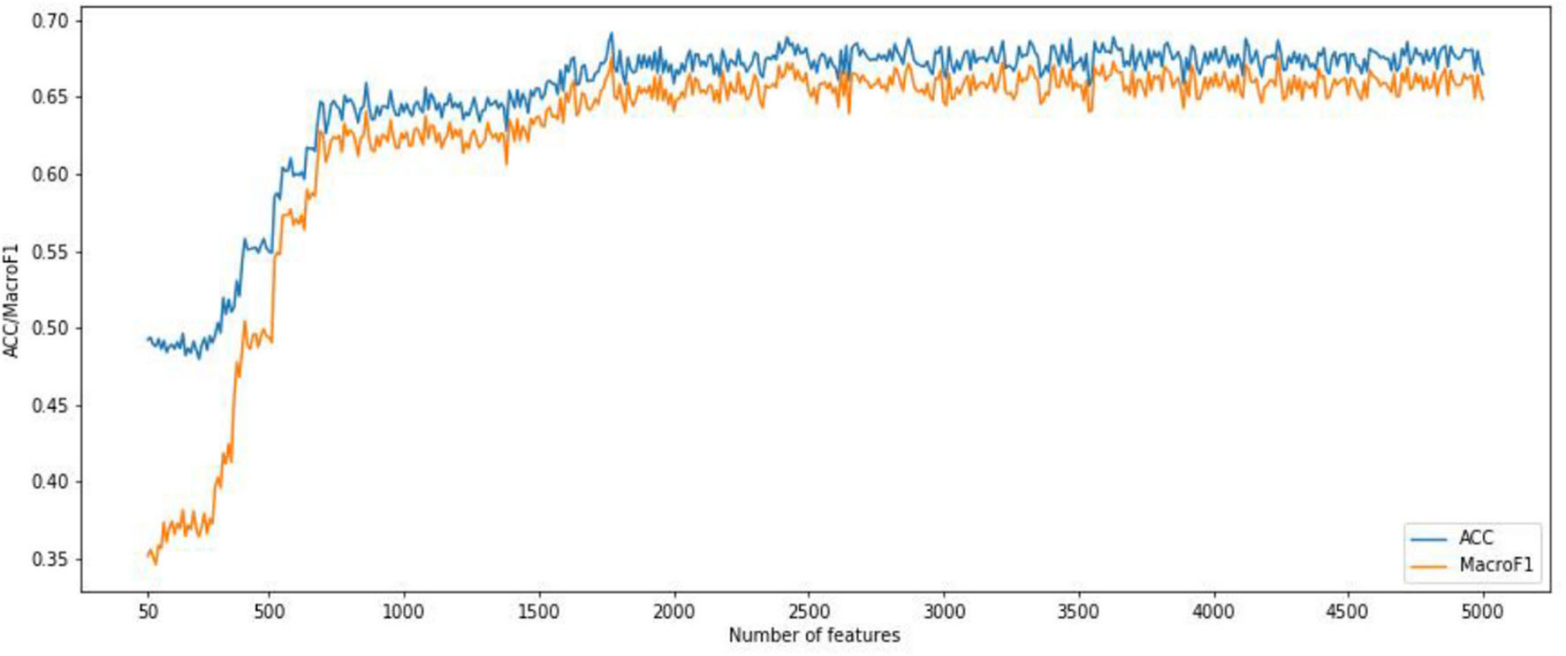
Incremental feature selection (IFS) curves derived from the IFS method and RF algorithm. IFS curve with *X*-values from 50 to 5,000.

We also implemented the RF algorithm to construct a classifier on the CNV features subset obtained from the IFS method and evaluate each classifier through a 10-fold cross-validation test. Since the fast speed of RF method, which promised all CNV feature sets were tested. In order to compare the classification feature selection results, the IFS curves of accuracy and macro F1 were plotted in Figure 4. It can be seen that the optimal accuracy value is 0.689 and the macro F1 is 0.667 when using the first 1,693 features in the CNV feature list. Therefore, the first 1,693 features and RF algorithms can construct the best RF classification model. It can be seen that the accuracy and macro F1 obtained by the best RF classifier are much lower than those obtained by the best SNN-based classification model. That means our SNN-based model is effective in pan-cancer classification analysis.

## Discussion

**Figure 5:**
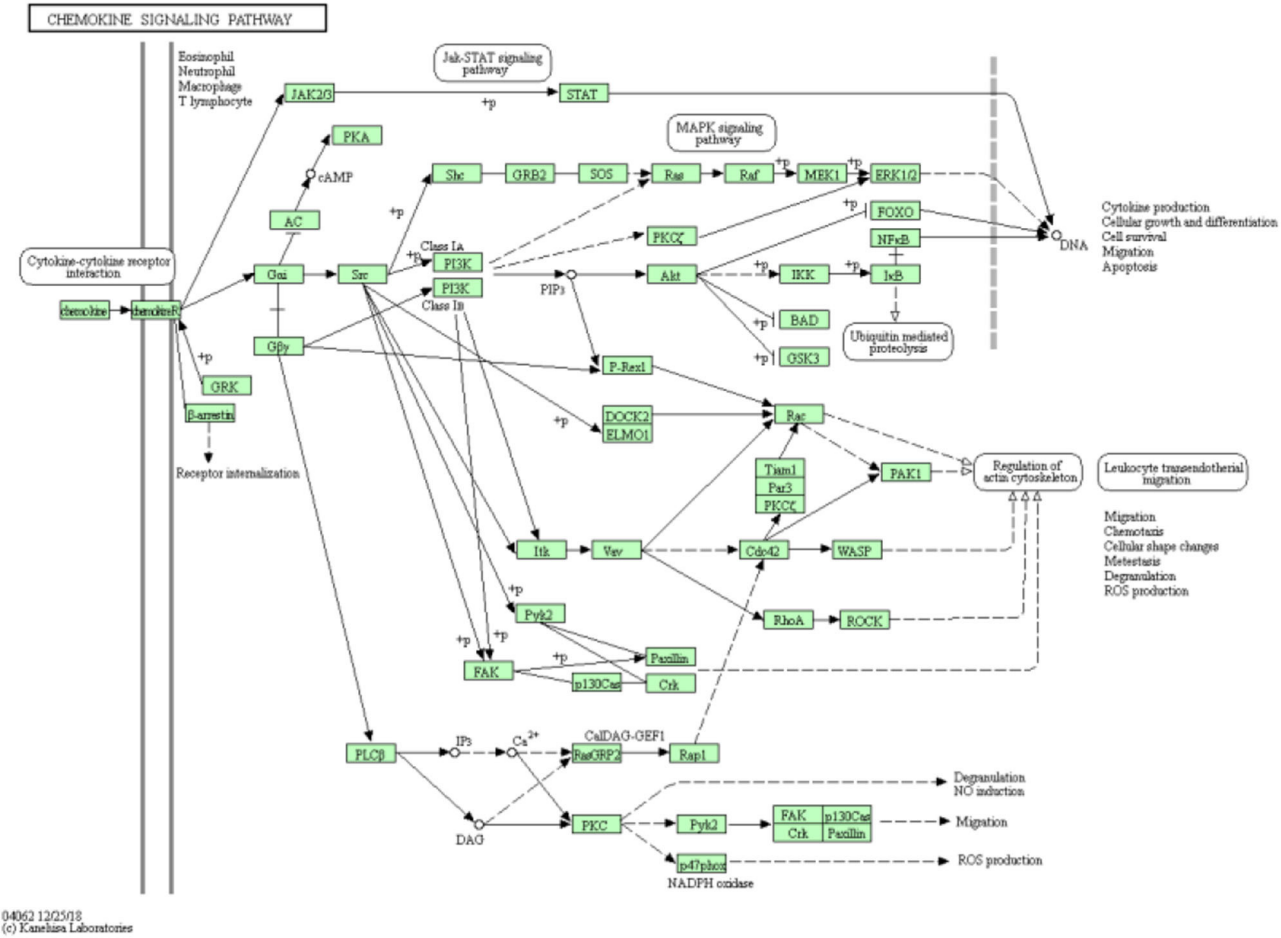
Chemokine signaling pathway from KEGG has the highest counts for selected feature genes.

DNA copy number variation is a straight-forward mechanism, which provides insight into genomic instability and structural dynamism in cancer researches. We applied Kyoto Encyclopedia of Genes and Genomes (KEGG) pathway enrichment analysis to the first 200 selected features and checked whether these were significant pathway information as shown as Figure 5. The highest counts are on the Chemokine signaling pathway, where chemoattractant proteins play an important role in controlling leukocyte migration during development, homeostasis, and inflammation. These processes are closely related to the occurrence and development of various cancers.

## Conclusions

In this study, we use machine learning method for CNV-based pan-cancer classification. Considering the high dimension of data, MCFS and IFS are used to classify four different cancer patients effectively. And the feature subsets generated from IFS method are classified by integrating SNN method. Comparison experiments show that our SNN-based classification method has significant advantages over random forest in cancer classification. We demonstrate the advantages and potential of this method for copy number variant data. We suggest that this model can be extended and transferred to other pan-cancer classification fields. For future research, we will improve the models of other complex and large-scale data and expand our training data sets to further improve classification results.

## Data Availability Statement

The data and code are available at https://github.com/KohTseh/CancerClassification.

## Author Contributions

JL and QX leaded the method application, experiment conduction, the result analysis, and drafted the manuscript. QX and MW participated in the data extraction and preprocessing. TH and YW provided theoretical guidance and the revision of this paper. All authors contributed to the article and approved the submitted version.

## Conflict of Interest

The authors declare that the research was conducted in the absence of any commercial or financial relationships that could be construed as a potential conflict of interest.
